# A novel application of stellate ganglion block to improve sleep: a systematic review and meta-analysis

**DOI:** 10.3389/fpsyt.2026.1753003

**Published:** 2026-03-30

**Authors:** Jie Wu, Diaofeng Zhang, Zixiao Liu, Yihan Yang, Zhipeng Wang, Ruifang Pu, Wei Deng, Jiale Wang, Guocan Shen, Chao Li, Wenting Tao, Min Zhao, Liming Cheng, Jiangang Li

**Affiliations:** 1 Department of Anesthesiology, Qujing Central Hospital of Yunnan Province, Qujing, Yunnan, China; 2 Department of Healthcare Security, Qujing Central Hospital of Yunnan Province, Qujing, Yunnan, China; 3 Department of Anesthesiology, School of Clinical Medicine, Dali University, Dali, Yunnan, China; 4 Department of Orthopedics, Qilin District People’s Hospital, Qujing, Yunnan, China; 5 Department of Anesthesiology, Kunming Children’s Hospital, Kunming, Yunnan, China

**Keywords:** anxiety, meta-analysis, psychological health, sleep disorders, stellate ganglion block

## Abstract

**Background:**

Sleep is essential for optimal health; however, the prevalence of poor sleep, characterized by disrupted patterns and qualities, adversely affects psychological well-being and cognitive function. This issue is prevalent, yet it is frequently underdiagnosed and inadequately managed. Current therapeutic options exhibit notable limitations. The Stellate Ganglion Block (SGB) may alleviate sleep disturbances through various physiological mechanisms; however, its safety and efficacy remain subjects of ongoing debate. This meta-analysis systematically evaluates the efficacy of SGB using objective metrics to provide high-quality clinical evidence and to inform future research directions.

**Methods:**

Literature searches were conducted in PubMed, Web of Science, Embase, the Cochrane Library, OVID, and Google Scholar from database inception to June 2025. Analyses were performed with RevMan 5.4 software, and the study was registered on PROSPERO (CRD420251040732) .

**Results:**

Seven studies met the inclusion criteria. In comparison to the control group, patients who received the SGB demonstrated a statistically significant increase in total sleep time (WMD: 60.86; 95% CI, 38.05 to 83.66; P < 0.001). Additionally, these patients exhibited lower scores on the Pittsburgh Sleep Quality Index (PSQI) (WMD: -1.22; 95% CI, -1.80 to -0.65; P < 0.001), reduced sleep onset latency (WMD: -1.59; 95% CI, -2.48 to -0.69; P < 0.001), and enhanced deep sleep quality, as indicated by lower scores on the deep sleep quality assessment (WMD: -1.42; 95% CI, -1.95 to -0.89; P < 0.001).

**Conclusion:**

The SGB shows promise in alleviating sleep problems. However, a lack of high-quality studies, insufficient long-term follow-up, and incomplete participant demographic data limit the existing evidence. There is a pressing need for extended follow-up and multicenter randomized controlled trials (RCTs). Future research should explore the potential synergies between stellate ganglion block and cognitive behavioral therapy (CBT-I) for sleep disorder to assess the sustainability of its efficacy and to expand the population that may benefit from this intervention.

**Systematic Review Registration:**

https://www.crd.york.ac.uk/prospero/, identifier CRD420251040732.

## Introduction

Sleep is a vital physiological process essential for overall health and well-being, playing a critical role in the normal functioning of various bodily systems. Poor sleep, characterized by disturbances in normal sleep patterns (1), manifests as dissatisfaction with either the quality or duration of sleep, difficulties in initiating or maintaining sleep, and subsequent daytime distress or functional limitations (2–4). A meta-analysis involving 948,882 participants found that the global pooled prevalence of insomnia among the general public was 29.7% (5). The pathophysiology of poor sleep is complex, involving dysregulation of circadian rhythms, homeostatic sleep drive, neurotransmitter systems, and neuroendocrine pathways (6). Following a comprehensive assessment of patients utilizing tools such as the Montreal Cognitive Assessment (MoCA) and the Social Functioning Questionnaire (SFQ), it was determined that these disruptions have a detrimental impact on psychological well-being and cognitive functions, including concentration, attention, and memory, as well as social functioning (7, 8). Despite its prevalence of up to 50% in primary care settings, poor sleep often remains underdiagnosed and inadequately managed, presenting a significant clinical challenge. This is primarily due to healthcare providers’ insufficient awareness, limited access to specialized diagnostic tools, and the complex interactions between poor sleep and coexisting medical or psychiatric conditions (1, 9). Furthermore, poor sleep may occur independently or in conjunction with various medical and mental health disorders, such as hypertension, type 2 diabetes, coronary artery disease, generalized anxiety disorder (GAD), and major depressive disorder (MDD). When left untreated, it serves as both a risk factor for developing comorbidities and an exacerbating factor for existing conditions (1, 10).

Various treatments for improving sleep include pharmacological interventions, physical therapies, traditional Chinese acupuncture, psychological treatments, and CBT-I (1, 2, 11). However, the prolonged use of sedative-hypnotics is associated with adverse effects, such as dizziness, asthenia, drug dependence, addiction, and withdrawal symptoms (2, 12, 13). Furthermore, the efficacy of physical therapy, acupuncture, and psychological interventions in alleviating sleep disturbances remains inconclusive, with a notable risk of recurrence (11, 14). In 2016, the American College of Physicians recommended CBT-I as the primary intervention for insomnia due to its favorable safety profile and efficacy (14). A meta-analysis conducted in 2019 confirmed the sustained effectiveness of CBT-I at three, six, and twelve months compared to no treatment; however, the clinical benefits in the active treatment group appear to diminish over time (15).

The stellate ganglion typically consists of the inferior cervical sympathetic ganglia (C7 to C8) and the superior thoracic sympathetic ganglia (T1). In approximately 80% of individuals, the inferior cervical and first thoracic ganglia merge to form the stellate ganglion (16). The administration of local anesthetics around the ganglion effectively interrupts the cervical sympathetic chain, thereby regulating autonomic tone in the affected regions (17). Recent studies advocate for the use of SGB in managing conditions such as post-traumatic stress disorder (18–20), cardiac arrhythmias (16, 21, 22), and hot flashes associated with cancer therapy and menopause (23–25).

SGB is suggested to enhance sleep regulation through various physiological mechanisms. It is theorized to reduce sympathetic hyperactivity by impeding nerve conduction at the stellate ganglion, leading to improved cerebral blood flow (26), synchronized melatonin secretion rhythms (12, 27), and suppression of sympathetic nerve excitation (28). These physiological adjustments are believed to ameliorate sleep problems (29–31). While controversy persists regarding the safety and efficacy of SGB for improving sleep quality (32, 33), existing systematic reviews have explored its efficacy in treating postoperative sleep disorders (34). However, this meta-analysis advances the discussion by focusing on the quantitative synthesis of clinical efficacy, incorporating objective parameters such as total sleep time and the PSQI. By critically evaluating the relevant literature on SGB’s role in managing sleep problems, this study aims to resolve lingering uncertainties, provide reliable evidence-based support for the clinical application of this intervention, offer substantiated evidence for clinical recommendations, and identify key areas for future investigation.

## Material and methods

This study followed PRISMA guidelines for both execution and reporting (35), and had been registered with PROSPERO(CRD420251040732).

### Search strategy

A comprehensive search was conducted across multiple databases, including PubMed, Web of Science, Embase, Cochrane Library, OVID, and Google Scholar, from their inception until June 2025. Additionally, a manual search of the bibliographies from identified review articles, original studies, and conference abstracts was performed. The search methodology strictly adhered to the PRISMA (Preferred Reporting Items for Systematic Reviews and Meta-Analyses) guidelines, with outcomes assessed using standardized criteria. For instance, the specific search strategy employed in PubMed is detailed in **Table 1**, which outlines the combinations of keywords and the filters applied.

**Table 1:** The detailed search strategy.

Search	PUBMED
#1	Search: (Sleep Initiation and Maintenance Disorders[MeSH Terms]) OR (((((((((((((((((((((((((((DIMS (Disorders of Initiating OR Maintaining Sleep)) OR (Disorders of Initiating OR Maintaining Sleep)) OR (Sleeplessness)) OR (Insomnia Disorder)) OR (Insomnia Disorders)) OR (Insomnia)) OR (Insomnias)) OR (Chronic Insomnia)) OR (Insomnia, Chronic)) OR (Early Awakening)) OR (Awakening, Early)) OR (Nonorganic Insomnia)) OR (Insomnia, Nonorganic)) OR (Primary Insomnia)) OR (Insomnia, Primary)) OR (Psychophysiological Insomnia)) OR (Insomnia, Psychophysiological)) OR (Rebound Insomnia)) OR (Insomnia, Rebound)) OR (Secondary Insomnia)) OR (Insomnia, Secondary)) OR (Sleep Initiation Dysfunction)) OR (Dysfunction, Sleep Initiation)) OR (Dysfunctions, Sleep Initiation)) OR (Sleep Initiation Dysfunctions)) OR (Transient Insomnia)) OR (Insomnia, Transient))
#2	Search: (stellate ganglion [MeSH Terms]) OR (((((((((Ganglion, Stellate) OR (Stellate Ganglia)) OR (Ganglia, Stellate)) OR (Ganglia, Stellate)) OR (Stellate Ganglia)) OR (Cervicothoracic Ganglia)) OR (Ganglia, Cervicothoracic)) OR (Cervicothoracic Ganglion)) OR (Ganglion, Cervicothoracic))
#3	#1 AND #2

### Inclusion criteria

(1) Patients underwent SGB; (2) Adequate data for meta-analysis was available; (3) Included RCTs or retrospective studies.

### Data extraction

(1) Excluded animal models, case reports, case series; (2) Studies using other treatment modalities in intervention or control groups; (3) Unpublished studies; (4) Challenges in obtaining relevant data.

### Study selection

The literature underwent a rigorous screening process utilizing EndNote reference management software to ensure accuracy and eliminate duplicates. Initially, two researchers scrutinized the titles to remove redundant entries, conference papers, protocols, reviews, and correspondence. The same two researchers then evaluated the abstracts to establish precise inclusion and exclusion criteria. Two investigators meticulously reviewed and approved the remaining literature for inclusion. Publications were independently assessed by two researchers, and selected studies were analyzed; those achieving unanimous agreement were included, with any discrepancies resolved by a third researcher.

### Data extraction

Data collection for study enrollment utilized a standardized eight-item data extraction form comprising author, country, year of publication, age, mean age, gender, weight, height, BMI, and outcomes.

### Potential for risk of bias

The Cochrane Collaboration’s risk of bias framework was employed to assess trial quality, with each domain rated as ‘low risk,’ ‘unclear risk,’ or ‘high risk’ (36).

### Analyzing data

The meta-analysis was conducted using RevMan 5.4 software. Statistical heterogeneity was assessed with Higgins I², indicating the percentage of total variation among trials. A fixed-effect model (Mantel-Haenszel method) was used for studies with an I² below 50%, while a random-effects model (Der Simonian-Laird) was applied to those with significant heterogeneity. Publication bias was examined through funnel plot visual inspection, complemented by Begg’s and Egger’s tests for asymmetry, with analyses performed using Stata 15.1 software.

## Results

### Characteristics of included trials

**Figure 1:**
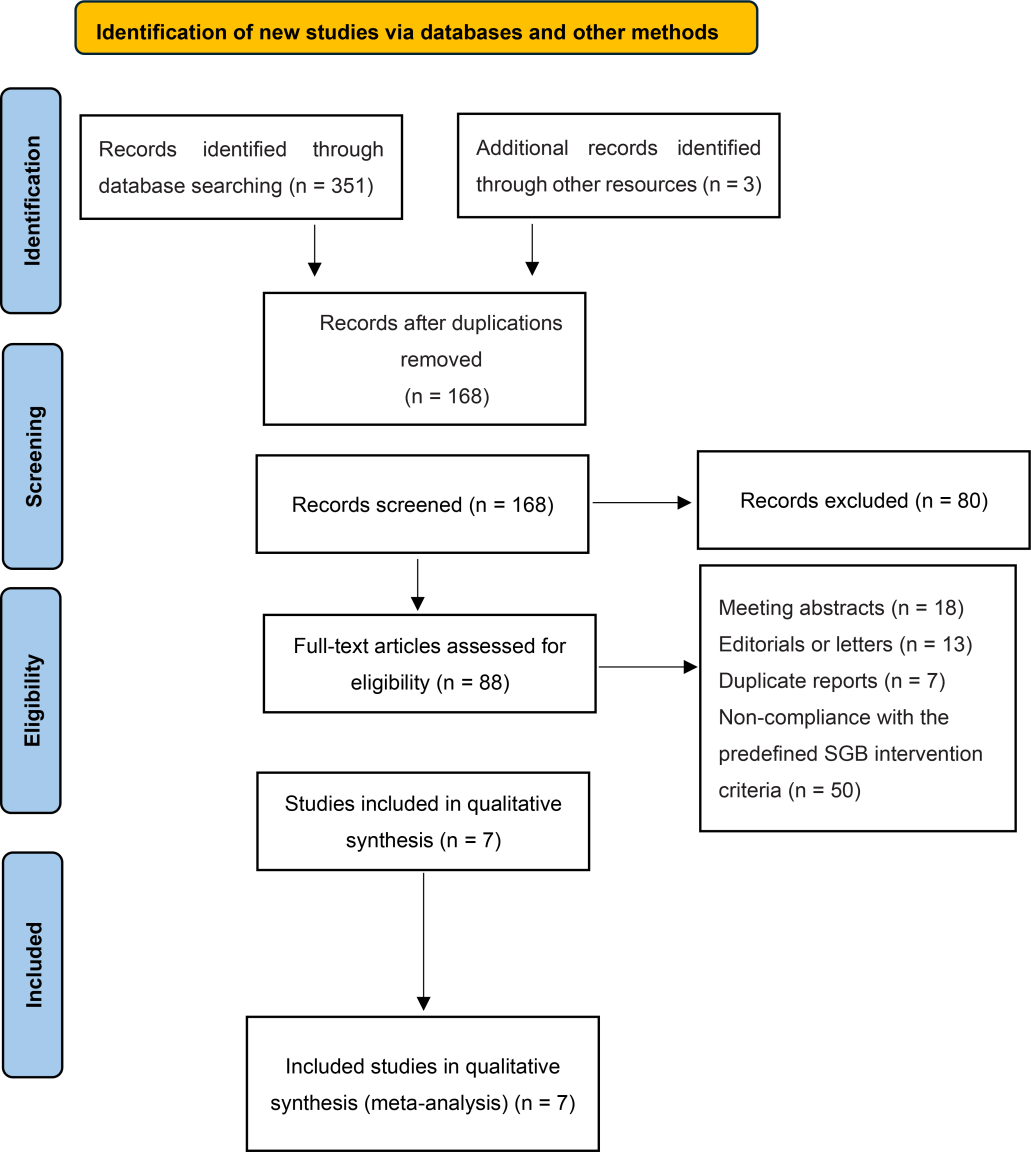
Flow diagram of literature selection.

The study selection process adhered to the guidelines outlined in the Preferred Reporting Items for Systematic Reviews and Meta-Analyses (PRISMA). A total of 351 records were retrieved from electronic databases, with an additional three studies identified through manual reference screening. After eliminating 168 duplicate records, 200 unique studies were screened based on their titles and abstracts. Subsequently, 88 articles underwent a full-text review. Following a thorough assessment, 81 studies were excluded for reasons such as being conference abstracts, lacking adequate outcome data, or not meeting the predefined SGB intervention criteria. Ultimately, seven studies involving 440 patients met the eligibility criteria for inclusion in this meta-analysis, as illustrated in the flow diagram of literature selection in **Figure 1** and detailed in **Table 2**.

**Table 2:** Characteristics of the included studies.

Author	Country	Year	Age (years)	Total/male/female	Weight(kg)	Height(cm)	BMI	AE	Control OM	Outcome
Xudong Ding (11)	China	2017	S:56.54 ± 9.23 C:55.62 ± 8.35	S:23/10/13 C:22/9/13	NA	NA	NA	Non-occurred	No treatment	PSQI TST deep sleep quality score Time for falling asleep
Poupak Rahimzadeh (23)	Iran	2018	S:34.80 ± 5.317 C:33.85 ± 5.566	S:20/0/20 C:20/0/20	S:73.20 ± 7.164 C:75.15 ± 7.132		S:27.10 ± 4.778 C:27.00 ± 4.712	Non-occurred	No treatment	PSQI
Caineng Wu (20)	China	2019	S:61.4 ± 8.9 C:59.6 ± 7.8	S:43/23/20 C:44/28/16	S:68.0 ± 9.9 C:65.9 ± 8.7	S:166.5 ± 8.1 C:165.1 ± 7.6	NA	Non-occurred	No treatment	TST
Decai Luo (37)	China	2023	S:52.87 ± 9.69 C:51.47 ± 8.89	S:30/19/11 C:30/18/12	S:64.07 ± 9.73 C:68.20 ± 10.33	S162.86 ± 7.38 C:162.83 ± 8.00	NA	Non-reported	No treatment	deep sleep quality score
Shiting Yan (38)	China	2023	S:69.5 ± 3.98 C:69.90 ± 4.53	S:20/12/8 C:20/11/9	S:65.75 ± 10.20 C:64.88 ± 11.03	S:166.60 ± 8.95 C:164.70 ± 8.74	NA	Non-reported	No treatment	PSQI TST
Yu Liu (39)	China	2023	S:33.5 ± 2.1 C:34.4 ± 1.9	S:20/8/12 C:20/10/10	NA	NA	S:21.97 ± 1.8 C:23.45 ± 2.1	Slight	Saline injection	deep sleep quality score
			S:33.5 ± 2.1 C:31.9 ± 2.7	S:20/8/12 C:20/8/12	NA	NA	S:21.97 ± 1.8 C:23.21 ± 1.9	Mild	Saline injection	deep sleep quality score
			S:33.5 ± 2.1 C:35.6 ± 5.4	S:20/8/12 C:20/11/9	NA	NA	S:21.97 ± 1.8 C:22.19 ± 2.0	Severe	Saline injection	deep sleep quality score Time for falling asleep
Ruizhi Yang (30)	China	2023	S:47.72 ± 7.90 C:48.61 ± 8.19	S:25/0/25 C:23/0/23	NA	NA	S:23.87 ± 3.24 C23.03 ± 2.98	Non-occurred	No treatment	TST

Kg, kilogram; cm, centimeter; TST, Total Sleep Time; PSQI, Pittsburgh Sleep Quality Index; BMI, Body Mass Index; SGB, Stellate Ganglion Block; OM, Operational methods; AE: adverse events.

### Quality assessment

**Figure 2:**
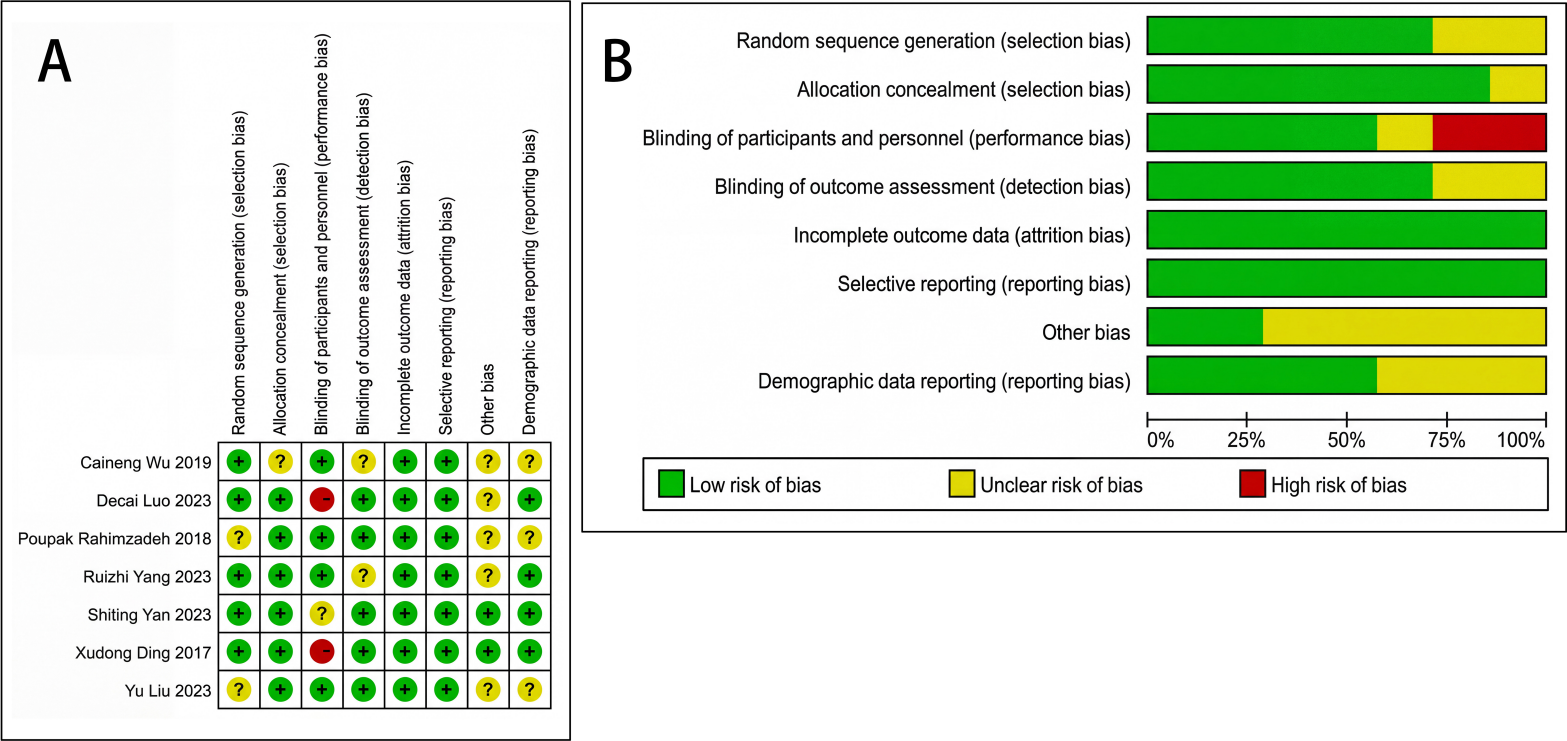
Analysis of bias risk for the included studies. **(A)** A summary of bias risk evaluating the authors’ assessments of each bias risk category for every included study. **(B)** Risk of bias graph illustrating the authors’ evaluations of each risk of bias criterion represented as percentages across all included studies.

The quality of the included studies was assessed using the Cochrane tool. **Figure 2A** summarizes the risk of bias ratings for each study, while **Figure 2B** illustrates the percentage distribution of these ratings across all bias domains.

### Publication bias and sensitivity analysis

The funnel plot analysis revealed symmetrical distributions for the majority of outcomes, as illustrated in the supplementary figures (**Supplementary Figures 2**, **5**). Further statistical evaluations, including Begg’s and Egger’s tests, confirmed the absence of significant publication bias, with all p-values exceeding.05 (see **Supplementary Table 1** in the **Supplementary Materials**). Sensitivity analyses highlighted the consistent reliability of the primary results, indicating that no individual study substantially influenced the aggregated effect sizes (**Supplementary Figures 6**, **9**).

### Meta-analysis results

#### Primary outcomes

**Figure 3:**
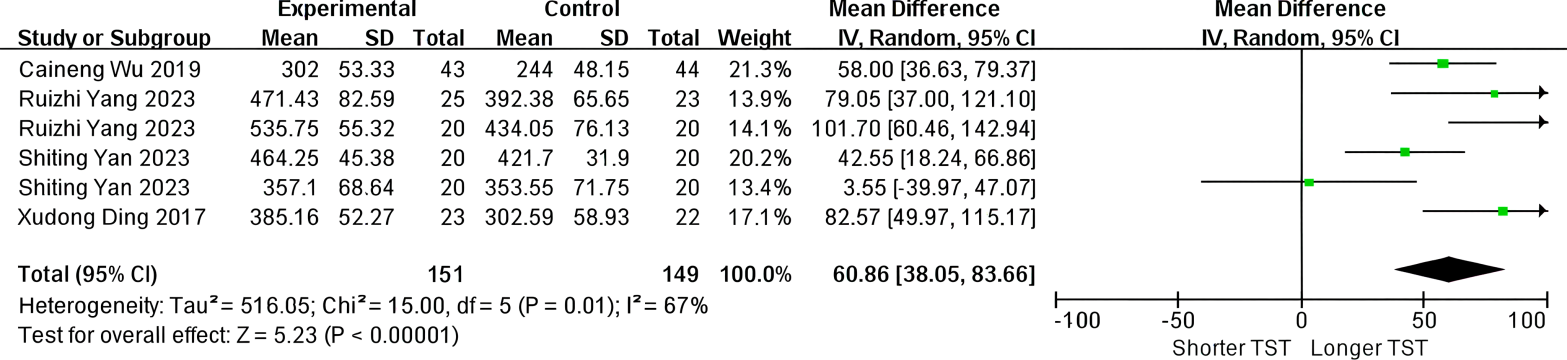
Forest plot of TST.

The analysis comprised four trials involving a total of 300 patients, investigating the impact of SGB treatment on total sleep time (TST). Due to substantial heterogeneity (P = 0.01, I²=67%), a random-effects model was employed for statistical evaluation. The findings revealed a significant increase in TST within the SGB group compared to the control group (WMD: 60.86; 95% CI, 38.05 to 83.66; P<0.001, **Figure 3**).

#### Results of subgroup analysis

We conducted subgroup analyses to explore the sources of heterogeneity, specifically examining factors such as postoperative day 1, postoperative days 2-3 (referred to as ‘Others’), and non-surgical treatment. The results indicated that, compared to the control group, the SGB group demonstrated a significantly elevated TST on postoperative day 1 (WMD: 70.68; 95% CI, 48.36 to 101.00; P < 0.001). However, no statistically significant difference was observed in the ‘Others’ group (WMD: 27.40; 95% CI, -9.86 to 64.65; P ≤ 0.15), suggesting that the long-term effects of SGB may contribute to the observed heterogeneity (Supplementary Figures, **Supplementary Figure 1**).

#### Secondary results

**Figure 4:**
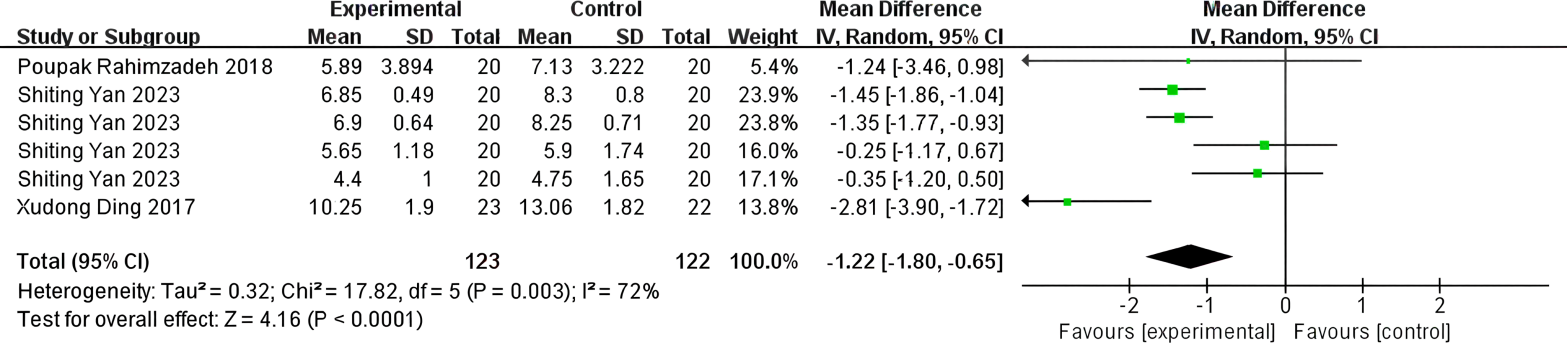
Forest plot of PSQI.

**Figure 5:**
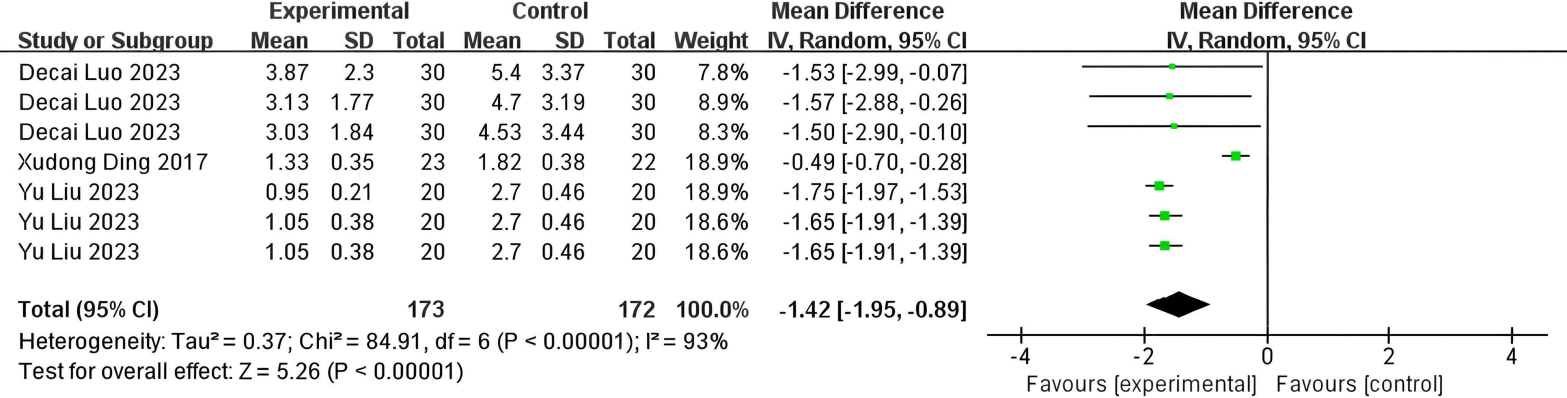
Forest plot of deep sleep quality score.

**Figure 6:**
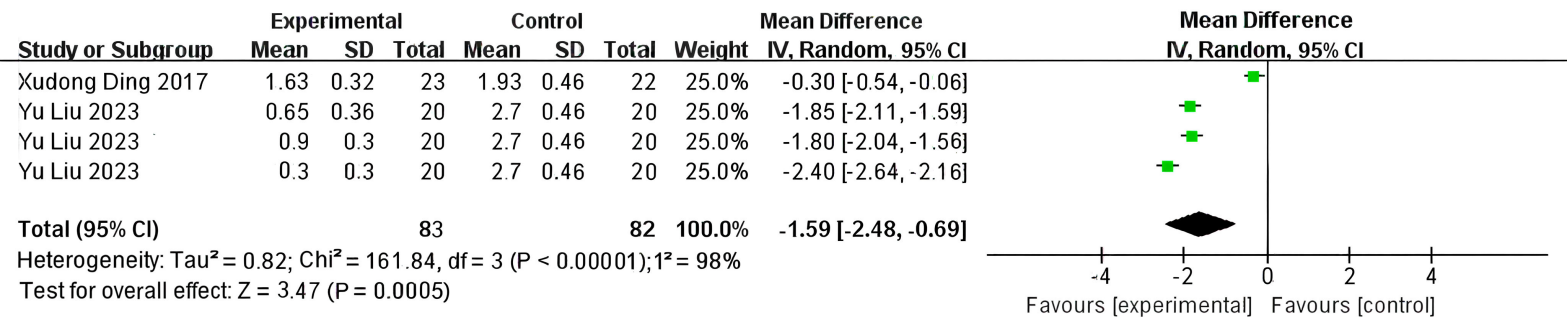
Forest plot of time for falling asleep.

The PSQI was assessed in 245 patients (P = 0.003, I²=72%). Analyses using a random-effects model revealed a significant difference between the SGB and control groups, with the PSQI being significantly lower (indicating better sleep quality) in the SGB group compared to the control group (WMD: -1.22; 95% CI, -1.80 to -0.65; P<0.001, **Figure 4**). A comparative analysis of Deep Sleep Quality Scores from 390 patients (P<0.001, I² = 93%) revealed that the SGB group exhibited significantly lower scores than the control group (WMD: -1.42; 95% CI, -1.95 to -0.89; P<0.001, **Figure 5**). Among these patients, 165 provided data regarding their Time for Falling Asleep. Due to substantial heterogeneity (I²=98%, P<.001), a random-effects model was employed, indicating that the SGB group experienced a faster sleep onset compared to the control group (WMD: -1.59; 95% CI, -2.48 to -0.69; P<0.001, **Figure 6**). According to the standards established by the World Health Organization (WHO), the Chinese Sleep Research Society developed the ‘Sleep Quality Assessment Scale.’ This scale consists of five components: sleep onset latency, nocturnal awakenings, early awakenings, sleep depth, and dreaming status. Each element receives a score between 0 and 3, resulting in an overall score that varies from 0 to 15, where higher scores reflect worse sleep quality. Specifically, a total score of less than 4 signifies satisfactory sleep quality, scores between 4 and 6 indicate poor quality, and scores above 6 reflect very poor quality, which may adversely affect both physical and mental health (37).

## Discussion

Poor sleep is influenced by a complex interplay of genetic, environmental, and behavioral factors, with a notably higher prevalence among females, adults suffering from mental or medical conditions, and individuals aged 45 or older, particularly during the perimenopausal and postmenopausal phases (40–42). Such disturbances severely impair both physical and psychological health, leading to cognitive deficits, memory issues, anxiety, and depression (26), and exhibit reciprocal relationships with cardiometabolic disorders (e.g., hypertension, diabetes, coronary artery disease) by disrupting cardiovascular regulation, increasing sympathetic activity, and elevating the risk of arrhythmias (43, 44). Moreover, poor postoperative sleep may precipitate arrhythmias (20), while enhanced sleep quality correlates with a reduced incidence of postoperative delirium (POD). From an economic perspective, insomnia imposes significant burdens, exemplified by Canada’s $1.9 billion in costs in 2021, which includes direct healthcare expenses and indirect productivity losses due to premature mortality (45). Additionally, untreated insomnia leads to increased absenteeism, decreased productivity, and higher accident risks (46). Despite these substantial impacts, chronic sleep disturbances are frequently overlooked in primary care settings (9, 47), with treatment delays often resulting from prolonged illness, poor treatment adherence (2, 48), concerns regarding medication side effects and dependency (49–51), and a global shortage of sleep specialists (2, 47). These factors culminate in suboptimal management, underscoring the urgent need for safe, minimally invasive, and reversible interventions.

This meta-analysis demonstrates that SGB effectively improves key sleep outcomes in patients, including increased total sleep time, enhanced sleep quality, and shortened sleep onset time, with statistical significance confirmed across trials. The stellate ganglion, a fusion of the lower cervical and first thoracic sympathetic ganglia, plays a pivotal role in regulating the autonomic nervous system (ANS). Growing evidence indicates that SGB suppresses excessive neural excitation, alleviates psychological stress, and balances sympathetic and parasympathetic tone—effects that directly enhance sleep quality (26, 52, 53). Crucially, sleep disorders are often driven by ANS dysregulation, characterized by sympathetic hyperactivity and parasympathetic hypofunction (54). To address this, SGB modulates both central and peripheral neural circuits to re-establish ANS homeostasis, while restoring endocrine balance and normalizing immune function. By inhibiting the sympathetic-adrenal axis and attenuating nociceptive signaling, SGB specifically targets the sympathetic overdrive that underlies sleep disturbances (20, 44). Liu et al. conducted a study involving 128 female patients diagnosed with generalized anxiety disorder and insomnia, demonstrating that SGB significantly reduced serum noradrenaline levels by 32%. Additionally, SGB improved heart rate variability, which serves as a surrogate indicator of autonomic balance, and was correlated with decreased PSQI scores (55).

Furthermore, SGB regulates autonomic activity and circadian rhythms through cervical sympathetic ganglion blockade. It corrects melatonin rhythm disruptions caused by sympathetic hypertonia (56) and enhances nocturnal serum melatonin levels (57), thereby directly promoting rapid sleep onset and sustaining optimal sleep (20). Melatonin, a key circadian hormone synthesized by the pineal gland, follows a distinct diurnal rhythm, with endogenous secretion peaking at night to synchronize the sleep-wake cycle (12, 58). While exogenous melatonin achieves supraphysiological plasma concentrations, the optimal dosing for insomnia remains controversial. Controlled trials in adults indicate modest reductions in sleep latency but minimal effects on sleep maintenance or total duration (12, 27, 59). Despite its growing use for pediatric sleep disorders, data regarding its efficacy and long-term safety are still limited (13). Notably, Uchida K et al. showed that SGB modulates pineal melatonin secretion, thereby re-establishing disrupted circadian rhythms (56). In alignment with this finding, Yang et al. reported that SGB significantly elevated serum melatonin levels and reduced interleukin-6 (IL-6) levels 24 hours post-intervention in postoperative female breast cancer survivors (30, 60). Proinflammatory cytokines, including IL-1, IL-6, and TNF-α, suppress rapid eye movement (REM) sleep (61, 62). Therefore, the dual capacity of SGB to normalize melatonin rhythms and regulate cytokine profiles likely underpins its efficacy in restoring physiological sleep architecture.

Cerebral blood flow (CBF) in humans is bidirectionally modulated by cervical sympathetic chain fibers and sleep-stage-specific neural activity (63, 64). CBF varies according to sleep stage and depth, being lowest during Non-Rapid Eye Movement (NREM) sleep and highest during REM sleep. This variation is attributed to regional cerebral processes that support functions such as modulating consciousness and processing emotions (65). In chronic insomnia disorder, patients exhibit elevated cortical CBF but reduced subcortical CBF (e.g., left putamen), with these changes correlating with symptom severity (66). Importantly, Wu CN et al. demonstrated that in patients undergoing thoracoscopic surgery, SGB improves sleep by inhibiting sympathetic innervation of the vertebrobasilar and posterior cerebral arteries, which enhances CBF in sleep-regulatory brain regions and promotes cortical recovery (20). This finding emphasizes the potential use of SGB as a non-systemic medication intervention to restore sleep-wake homeostasis.

In summary, SGB improves sleep via multiple mechanisms (67), as a reversible, minimally invasive intervention, it circumvents the systemic side effects and dependency risks associated with pharmacotherapies (68, 69), positioning it as a promising option for complex sleep disorder management. Compared to other non-pharmacological interventions, the SGB presents distinct advantages. Repetitive Transcranial Magnetic Stimulation (rTMS) requires prolonged sessions and expensive equipment (70), while Vagal Nerve Stimulation (VNS) is often limited by the necessity of invasive implantation or specific indications (16). Although acupuncture is regarded as safe, its effectiveness is compromised by high operator dependency and an unclear mechanism of action (66). CBT-I is widely recommended as a first-line treatment (14, 71–73). A double-blind RCT enrolled 128 patients with generalized anxiety disorder alongside sleep disturbances (excluding depression), randomly assigning them to receive either SGB (n=64, 4 ultrasound-guided 1% lidocaine injections) or conventional treatment (n=64, CBT-I + estazolam 1–2 mg/day) (55). Following a four-week period, the SGB treatment notably decreased the severity of anxiety, enhanced sleep quality, and adjusted neurotransmitter levels (lowered norepinephrine, increased serotonin and neuropeptide Y), indicating a possible mechanism related to the regulation of the sympathetic nervous system. Notably, the data from this RCT were excluded from the current meta-analysis due to its control arm comprising conventional treatment (CBT-I + estazolam). Given the first-line status of CBT-I and the demonstrated efficacy of SGB, future research should explore their combined synergistic effects. The investigation aims to determine whether this multimodal strategy produces superior outcomes and expands the pool of eligible beneficiaries.

Our findings must be interpreted within the context of the limitations inherent to this meta-analysis. Advancements in ultrasound technology have significantly improved its safety and success rates (74), with no serious complications reported in the studies included in this meta-analysis. However, Liu et al. observed that in the high-volume group (8 mL of 0.375% ropivacaine), local anesthetic diffusion to the common carotid artery region occurred more frequently, resulting in a significantly higher incidence of hoarseness compared to the other two groups (39). This indicates that drug volume may be a critical factor in triggering adverse reactions. Therefore, future research should focus on determining the optimal dosage for different patient populations to further enhance the clinical efficacy of SGB. According to the results of the subgroup analysis, the duration of the SGB may contribute to the heterogeneity observed in the TST. On the first postoperative day, SGB exhibited significant efficacy for TST; however, its long-term effects necessitate further investigation. This time-dependent efficacy pattern is likely associated with the pharmacokinetic characteristics of local anesthetics, such as lidocaine and ropivacaine, used in SGB. The neural blocking effects of these anesthetics typically last between 4 to 24 hours following a single injection. As the anesthetic dissipates, sympathetic tone gradually recovers, weakening the therapeutic effect on sleep duration. Future research should also examine whether SGB requires repeated administration and determine the appropriate interval between sessions.

All seven studies included in this research explicitly reported gender distribution data (see **Table 2**). However, none of the studies documented the participants’ menopausal status, which precludes targeted subgroup analyses that could examine gender differences and menopause-related factors more effectively. The current evidence exhibits geographic and cultural biases, as the majority of the included studies are sourced from China. This limitation restricts the generalizability of SGB across diverse populations, given the potential variability in treatment response and healthcare access. Furthermore, existing trials have predominantly concentrated on short-term outcomes. Data regarding long-term recurrence rates and sustained efficacy remain scarce; thus, longitudinal studies with extended follow-up (≥1 year) are essential to bridge this gap. Notably, significant heterogeneity was observed across certain outcomes, despite the application of random-effects models to account for inter-study variability. This was particularly evident in the secondary endpoints, where exceptionally high heterogeneity was noted for ‘Deep Sleep Quality Scores’ (I² = 93%) and ‘Time for Falling Asleep’ (I² = 98%). This inconsistency can largely be attributed to variations in the interventions of the control groups, which include both ‘no treatment’ and ‘saline injection.’ Furthermore, it may also stem from differences in study populations, such as mixed-sex versus female-only cohorts, as well as the absence of data on factors such as BMI and comorbidities. Due to the limited number of existing studies, no investigation into sources of heterogeneity was conducted for other indicators. To address these limitations, future research should prospectively collect comprehensive demographic and clinical data. This strategy will enable subgroup analyses of SGB efficacy, clarifying whether treatment benefits differ by sex or obesity status, and will offer essential insights for personalized clinical guidelines.

## Conclusion

This meta-analysis demonstrates that the stellate ganglion block (SGB) significantly improves key sleep metrics, including prolonged total sleep time and enhanced sleep quality, thereby highlighting its potential for treating sleep disorders. However, the evidence is constrained by a limited number of high-quality studies, inadequate long-term follow-up, and incomplete participant demographics. There is an urgent need for well-designed, prospective multicenter randomized controlled trials (RCTs) with extended follow-up to evaluate the sustained real-world efficacy of SGB. Future research should investigate the potential synergies between SGB and CBT-I to determine if a multimodal therapeutic approach produces better outcomes, broadens the applicable populations, and improves clinical practice.

## Data Availability

The original contributions presented in the study are included in the article/**Supplementary Material**. Further inquiries can be directed to the corresponding author.
