# Supplementary material for: A novel application of stellate ganglion block to improve sleep: a systematic review and meta-analysis
Source: Front Psychiatry. 2026 Mar 30;17:1753003. doi: 10.3389/fpsyt.2026.1753003 (PMC13071040; doi:10.3389/fpsyt.2026.1753003)
Supplement: Supplementary file 1 [file Supplementaryfile1.docx]

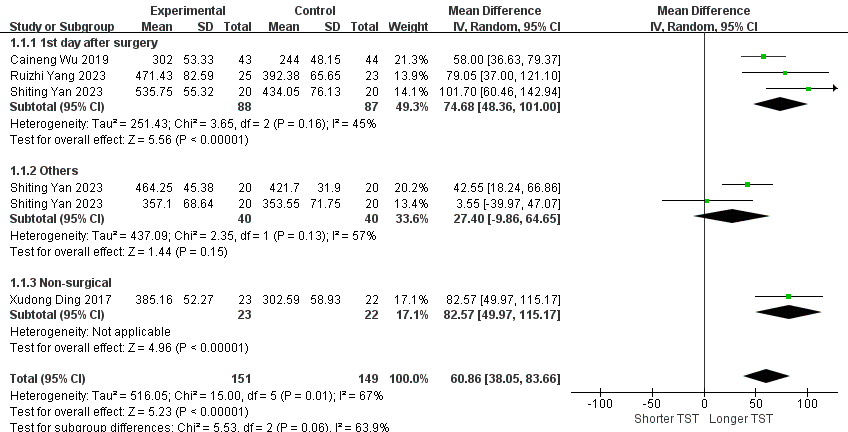


Figure S1: Subgroup of TST


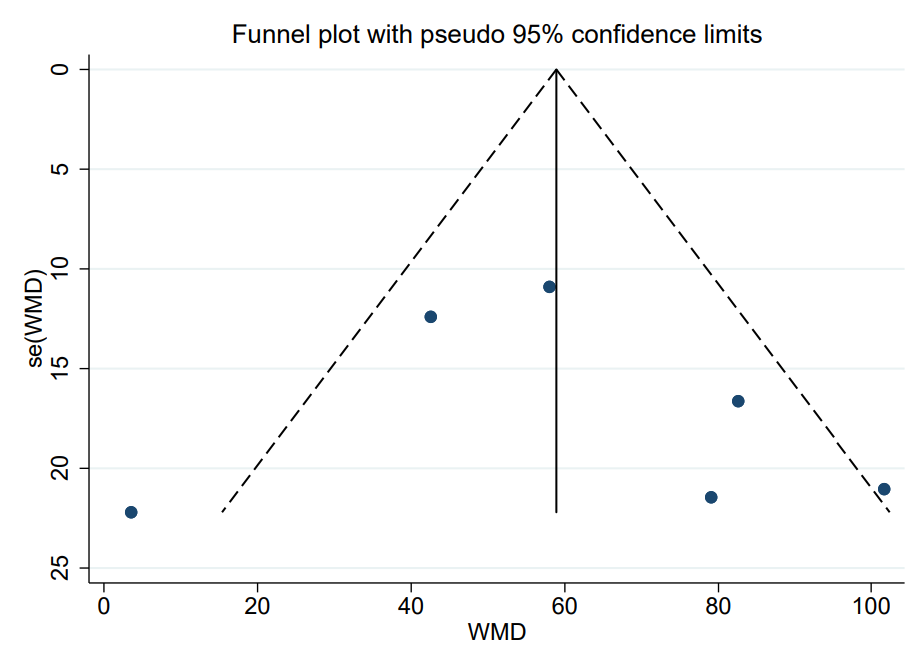


Figure S2: Funnel plot of TST.


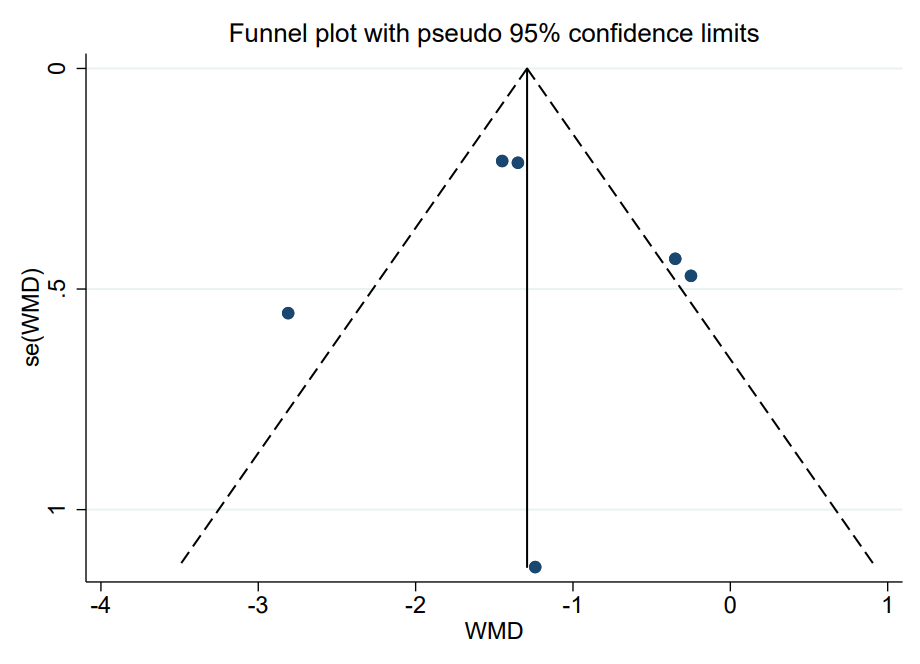
Figure S3: Funnel plot of PSQI.


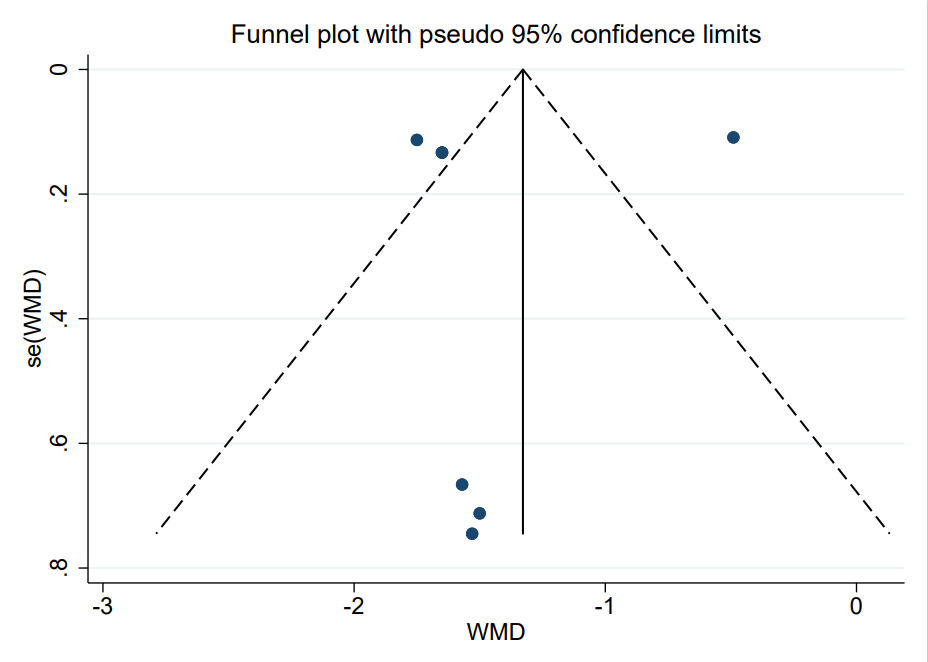


Figure S4: Funnel plot of deep sleep quality score.


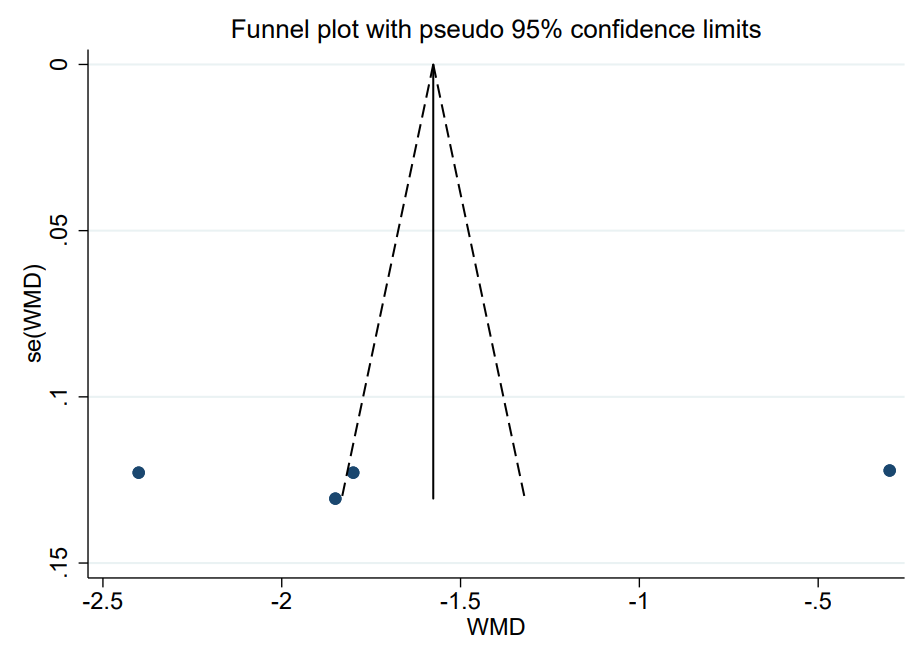


Figure S5: Funnel plot of time for falling asleep.

Figure S6: Sensitivity analysis of TST.

Figure S7: Sensitivity analysis of PSQI.

Figure S8: Sensitivity analysis of deep sleep quality score.

Figure S9: Sensitivity analysis of time for falling asleep.
