# Supplementary material for: A novel application of stellate ganglion block to improve sleep: a systematic review and meta-analysis
Source: Front Psychiatry. 2026 Mar 30;17:1753003. doi: 10.3389/fpsyt.2026.1753003 (PMC13071040; doi:10.3389/fpsyt.2026.1753003)
Supplement: Supplementary file 2 [file Supplementaryfile2.docx]

**GRADE assessment table**

| **Certainty assessment** | | | | | | | **№ of patients** | | **Effect** | | **Certainty** | **Importance** |
| --- | --- | --- | --- | --- | --- | --- | --- | --- | --- | --- | --- | --- |
| **№ of studies** | **Study design** | **Risk of bias** | **Inconsistency** | **Indirectness** | **Imprecision** | **Other considerations** | **TST** | **placebo** | **Relative (95% CI)** | **Absolute (95% CI)** |  |  |
| **TST** | | | | | | | | | | | | |
| 4 | randomised trials | not serious | not serious | not serious | serious | none | 151 | 149 | - | MD **60.86 higher** (38.05 higher to 83.66 higher) | ⨁⨁⨁◯ Moderate | CRITICAL |
| **PSQI** | | | | | | | | | | | | |
| 3 | randomised trials | not serious | not serious | not serious | not serious | none | 123 | 122 | - | MD **1.22 lower** (1.8 lower to 0.65 lower) | ⨁⨁⨁⨁ High | CRITICAL |
| **deep sleep quality score** | | | | | | | | | | | | |
| 3 | randomised trials | serious | not serious | not serious | not serious | none | 173 | 172 | - | MD **1.42 lower** (1.95 lower to 0.89 lower) | ⨁⨁⨁◯ Moderate | IMPORTANT |
| **Time for falling asleep** | | | | | | | | | | | | |
| 2 | randomised trials | not serious | not serious | serious | not serious | none | 83 | 82 | - | MD **1.59 lower** (2.48 lower to 0.69 lower) | ⨁⨁⨁◯ Moderate | IMPORTANT |

**CI:** confidence interval; **MD:** mean difference
