# Supplementary material for: A novel application of stellate ganglion block to improve sleep: a systematic review and meta-analysis
Source: Front Psychiatry. 2026 Mar 30;17:1753003. doi: 10.3389/fpsyt.2026.1753003 (PMC13071040; doi:10.3389/fpsyt.2026.1753003)
Supplement: Supplementary file 3 [file Table1.docx]

**Table S1 (Online Supplementary). Assessment for publication bias**

| Assessment for publication bias | | | | |
| --- | --- | --- | --- | --- |
|  | TST | PSQI | Deep sleep quality score | Time for falling asleep |
| P value for Begg's test | 0.851 | 0.573 | 0.368 | 0.279 |
| P value for Egger's test | 0.719 | 0.790 | 0.776 | 0.740 |

TST, Total Sleep Time; PSQI, Pittsburgh Sleep Quality Index.
